# Chronic alcohol overconsumption may alter gut microbial metabolism: a retrospective study of 719 ^13^C-D-xylose breath test results

**DOI:** 10.1080/16512235.2017.1301725

**Published:** 2017-03-17

**Authors:** Steinar Traae Bjørkhaug, Viggo Skar, Asle W. Medhus, Anita Tollisen, Jørgen G. Bramness, Jørgen Valeur

**Affiliations:** ^a^ Unger-Vetlesen Institute, Lovisenberg Diaconal Hospital, Oslo, Norway; ^b^ Department of Gastroenterology, Oslo University Hospital, Oslo, Norway; ^c^ Norwegian National Advisory Unit on Concurrent Substance Abuse and Mental Health Disorders (Norwegian Centre of Dual Diagnosis), Innlandet Hospital Trust, Brumunddal, Norway; ^d^ Norwegian Centre for Addiction Research, University of Oslo, Oslo, Norway

**Keywords:** Alcohol, breath tests, gut microbiota, malabsorption, xylose

## Abstract

**Objective**: Alterations of gut microbiota composition or function may participate in the pathophysiology of several diseases. We aimed to explore the effect of chronic alcohol overconsumption on gut microbial metabolism, as assessed by evaluating ^13^C-D-xylose breath test results.

**Materials and methods**: We investigated all ^13^C-D-xylose breath tests performed at Lovisenberg Diaconal Hospital during the years 2005 to 2011, using patient files for diagnosing the patients into one of three patient categories: alcohol overconsumption, coeliac disease and functional bowel disorder. In addition, a group of healthy controls was included. The time curves of ^13^CO_2_ excretion in breath samples were divided into two phases, evaluating small intestinal absorption (0–60 min) and colonic microbial metabolism (90–240 min), respectively.

**Results**: A total of 719 patients underwent ^13^C-D-xylose breath testing during the inclusion period. Thirty-five had a history of alcohol overconsumption, 66 had coeliac disease, and 216 had a functional bowel disorder, while 44 healthy controls were included for comparison. The alcohol overconsumption group had similar small intestinal phase results as the group of patients with untreated coeliac disease. During the colonic phase, the group of patients with alcohol overconsumption differed from all the other groups in terms of ^13^C-xylose recovery, with significantly less ^13^CO_2_ excretion compared to the other groups.

**Conclusion**: The results suggest that patients with a history of alcohol overconsumption suffer from both small intestinal malabsorption and impaired colonic microbial metabolism. The role of gut microbiota in chronic alcohol overconsumption should be investigated further.

## Introduction

Alcohol exerts several harmful effects on the gastrointestinal tract and associated glands.[1–3] During recent years, alterations of the gut microbiota and subsequent damage to hepatocytes have emerged as an important pathophysiological mechanism by which alcohol may contribute to hepatic cirrhosis and liver failure.[4] In particular, leakage of immuno-active endotoxins from the gut into the blood may be involved. A similar mechanism has recently been proposed as an explanation for the high incidence of anxiety and depression seen among patients with elevated alcohol consumption, and as a mechanism of alcohol craving following periods of detoxification.[5] Thus, investigation of the microbiota seems to be a rational approach for examining how alcohol affects the body.

The gut microbiota can be evaluated either by assessing its composition or by measuring its functions. The term microflora-associated characteristic (MAC) has been introduced to designate the latter approach, being defined as ‘the recording of any anatomical structure or physiological, biochemical, or immunological function in a macro-organism, which has been influenced by the microflora in either an anabolic or catabolic way’.[6] In the present study, we aimed to explore the effect of chronic alcohol overconsumption on gut microbiota function by assessing metabolism of xylose as a MAC, using ^13^C-D-xylose breath testing.

The D-xylose breath test has previously been used extensively as a tool to diagnose small intestinal bacterial overgrowth (SIBO), taking advantage of the microbial ability to metabolize D-xylose.[7] Furthermore, D-xylose is a pentose, of which around 70% of the ingested amount is passively absorbed in the small intestine. Thereafter, absorbed D-xylose is either metabolized by the liver into threitol and carbon dioxide, excreted in the urine, or excreted unchanged into the bile.[8–10] Using an isotope of carbon to mark the D-xylose, the amount of exhaled ^X^CO_2_ might be used as an expression of the effective absorption area of the small intestine, and thus be useful in diagnosing malabsorption. During later years, the ^13^C-D-xylose breath test has replaced the previously used ^14^C-D-xylose breath test for this purpose,[11] due to ^14^C being a radioactive isotope. Previous studies, applying administration of the ^14^C-isotope in patients with chronic alcohol overconsumption, have concluded that alcohol may cause malabsorption comparable to patients with untreated coeliac disease.[12] The gut microbiota is mainly located within the colon. As microbial metabolism of ^13^C-D-xylose produces ^13^CO_2_ in a similar manner as via hepatic metabolism,[13] and the test duration is 240 min, the ^13^CO_2_ excretion increases from the point when the test meal reaches the caecum. Hence, the ^13^C-D-xylose breath test may also be used as an instrument to investigate the gut microbial metabolism, during the colonic phase. In the present study, a consecutive material of ^13^C-D-xylose breath tests performed during the period 2005–2011 at Lovisenberg Diaconal Hospital was reviewed in order to explore whether chronic alcohol overconsumption may alter metabolism of D-xylose during the colonic phase, as a measure of gut microbial function.

## Materials and methods

### Study participants

Consecutive patients referred to ^13^C-D-xylose breath testing at Lovisenberg Diaconal Hospital (Oslo, Norway) during the years 2005–2011 were included. They were diagnosed based on clinical information from the referring doctor and the patients’ hospital file (including available blood chemistry results, biopsy reports and radiological examinations), by an experienced gastroenterologist, into the following groups: alcohol overconsumption, coeliac disease or patient controls. Patients with alcohol overconsumption were included based on confirmed anamnestic information regarding alcohol overconsumption, in amounts corresponding to several units per day for several years. In addition, these patients had blood samples showing one or more of the following: macrocytic anaemia (haemoglobin <11.7 g/100 ml in women or <13.4 g/100 ml in men; MCV>98 fl), thrombocytopenia (<145 x 10^9^ l^–1^), low sodium levels (<137 mmol l^–1^), elevated γGT levels (>45 U l^–1^), and elevated AST/ALT-ratio (>2 with elevated AST >35 U l^–1^). Regarding coeliac disease, the diagnosis was confirmed by duodenal biopsies. The patient control group consisted of patients who were diagnosed as having a functional bowel disorder, mainly irritable bowel syndrome (IBS). All patients were referred as part of routine investigation. In addition, 30 patients with coeliac disease and 29 patients with alcohol overconsumption were recruited as part of previous malabsorption studies.[11,14–16] The group of healthy controls were recruited from the hospital staff. Patients with a history of gastrointestinal surgery or overt clinical signs of liver failure were excluded from the study. The 348 patients not fulfilling the inclusion criteria were patients diagnosed into one of the following groups: inflammatory bowel disease (*n *= 23), treated coeliac disease (*n *= 55), SIBO (*n *= 4), food intolerances apart from coeliac disease (*n *= 47), borderline coeliac disease (*n *= 36) and ‘miscellaneous’ (*n *= 120). The miscellaneous group consisted of patients with diabetes mellitus (*n *= 23), iron deficiency (*n *= 26), thyroid disease (*n *= 37), cholecystectomy (*n *= 8), pernicious anaemia (*n *= 12), cancer (*n *= 9), osteoporosis (*n *= 5) and ‘various’ (*n *= 63). Within the latter group, consisting of patients with conditions such as sarcoidosis, microscopic colitis, PBC, Addison’s disease and suspected but unconfirmed IBD, no group consisted of more than three patients, and none of the patients fulfilled the criteria for the three study groups. Consequently, they were not included in the further analysis of the material. For the remaining 10 patients (20% male), we did not have sufficient information regarding their medical condition to diagnose them within the limits of this study. The study was carried out according to the Declaration of Helsinki and approved by the Regional Committee for Medical Research Ethics (REK Sør-Øst; reference number 2013/2357).

### ^13^C-D-xylose breath test

Tveito et al. [11] have previously validated the use of the test in diagnosing malabsorption. Briefly, the procedure is performed after an overnight fast, and starts with two basal samples before the test meal is consumed. The test meal consists of 100 mg of 99% ^13^C-D-xylose (Cambridge Isotope Laboratories, Cambridge, MA, USA) and 5 g of xylose dissolved in 250 ml of tap water. Breath samples are taken times two every 30 min during the test, collected from end-expirium through a straw into 12 ml exetainers (Labco Limited, High Wycombe, UK), with a total test duration of 240 min. A ^13^CO_2_: ^12^CO_2_ ratio is determined by gas chromatography and continuous flow isotope mass spectrometry (ABCA 20/20; Europa Scientific, Crewe, UK), expressed as percentage of ^13^C recovery per hour (% dose h^–1^), using formula from Scholler et al. [17]. CO_2_ production is assumed to be 300 mmol m^–^^2^, while body surface is calculated using weight-height formula from Haycock et al. [18]. In order to evaluate the small intestinal phase and the colonic phase, we used a standard oro-caecal transit time (OCTT) of 90 min, based on previous examinations on OCTT assessed with lactulose breath test.[19,20] For the analyses, we focused on the total amount of exhaled ^13^CO_2_. Hence, total area under the curve at 240 min subtracted area under the curve from zero to 90 min, was computed for all patients for the colonic phase. For the small intestinal phase, area under the curve for the first 60 min was computed for all patients.

### Statistical analyses

We applied SPSS version 17.0 for statistical analyses, using *t*-tests to compare differences between groups. All tests were two-tailed, and *p*-values less than 0.05 were set as a limit for statistical significance.

## Results

### Subject characteristics

A total of 719 patients underwent the ^13^C-D-xylose breath test during the inclusion period (Table 1), of whom 35 had a history of alcohol overconsumption (89% male; mean age 53.7; range 38–78 years). Sixty-six were diagnosed with coeliac disease (35% male; mean age 37.9; range 18–89 years), and 216 with IBS (31% male; mean age 34.4; range 13–78 years). Forty-four healthy controls (30% male; mean age 38.4; range 22–67 years) were included for comparison.

**Table 1.** Baseline characteristics of study participants recruited from a consecutive material of ^13^C-D-xylose breath test results, collected at Lovisenberg Diaconal Hospital from 2005 to 2011, with a total *n *= 719.

Group	Alcohol overconsumption	Coeliac disease	Patient controls	Healthy controls
N (%male)	35 (88%)	66 (35%)	216 (31%)	44 (30%)
Age (mean, range)	53.7 (38–78)	37.9 (18–89)	34.4 (13–78)	38.4 (22–67)
BMI	23.7 (15.4–44.8)	22.4 (17.0–35.9)	22.9 (15.9–36.1)	23.5 (19.3–32.0)

### ^13^C-D-xylose breath test results

**Figure 1.**
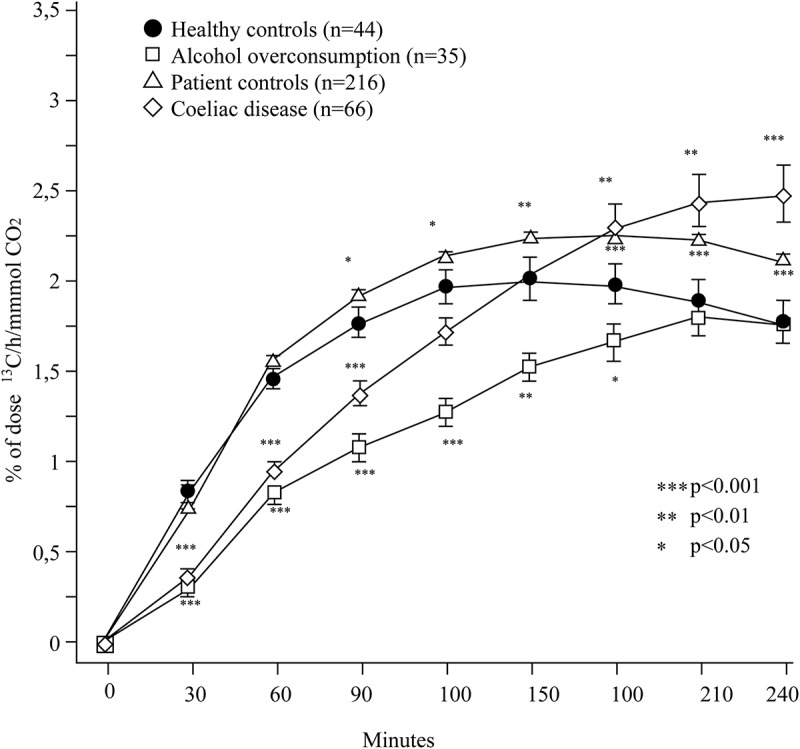
Excretion of ^13^CO_2_ after ^13^C-D-xylose ingestion in different patient groups. The y-axis represents amount (percentage) of test meal expired per hour, while the x-axis represents time (minutes). During the small intestinal phase of the test (0–60 min), the group of patients with alcohol overconsumption and coeliac disease are similar, collectively differing from the other two groups. During the colonic phase of the test (90–240 min), these two groups have a distinctly different pattern, the latter with a rebound phenomenon lacking in the prior group.

**Figure 2.**
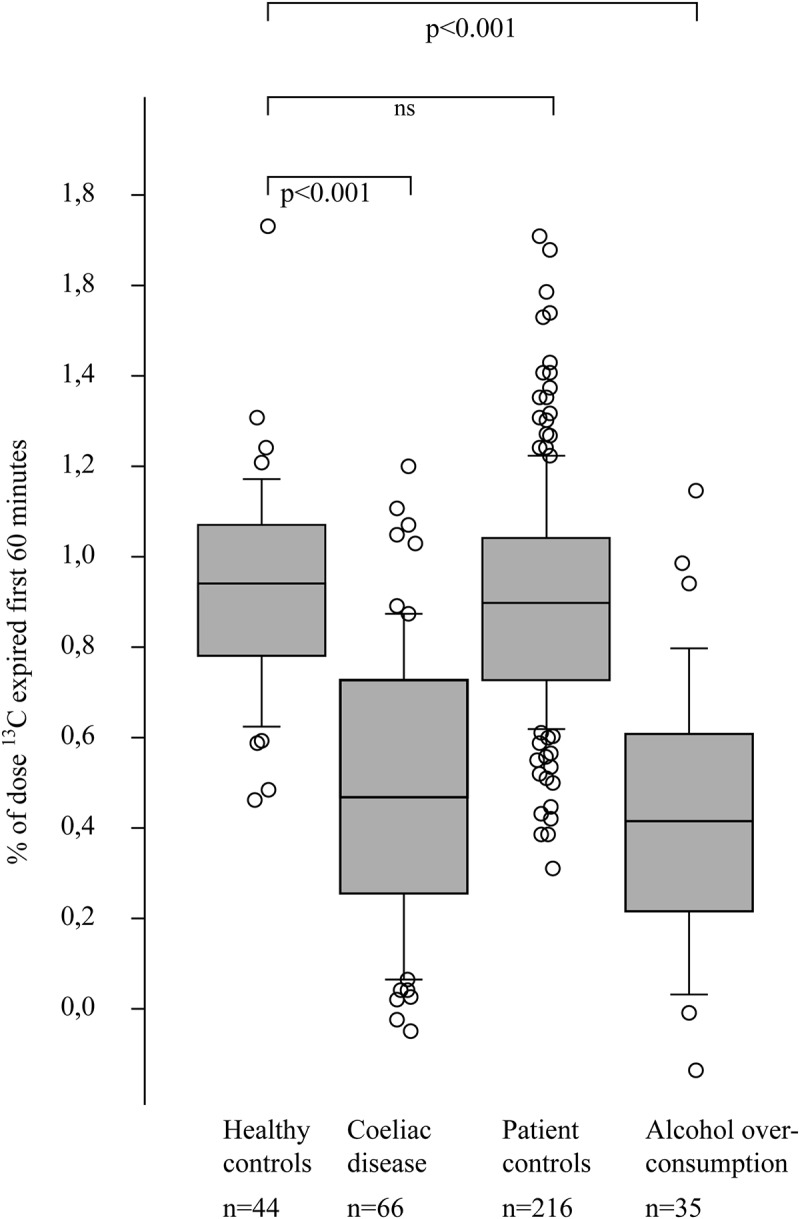
Box plot showing the small intestinal phase (0–60 min) of ^13^CO_2_ excretion after ingestion of ^13^C-D-xylose in different patient groups. The results are expressed as area under the curve (percentage of dose ^13^C-recovery) for the first 60 min of the test, hence showing total amount of ^13^C-recovery. Compared to healthy controls and patient controls, patients with untreated coeliac disease and patients with high alcohol consumption show a pattern suggestive of malabsorption. The differences are statistically significant with *p*-values <0.001 for both groups compared to healthy controls.

**Figure 3.**
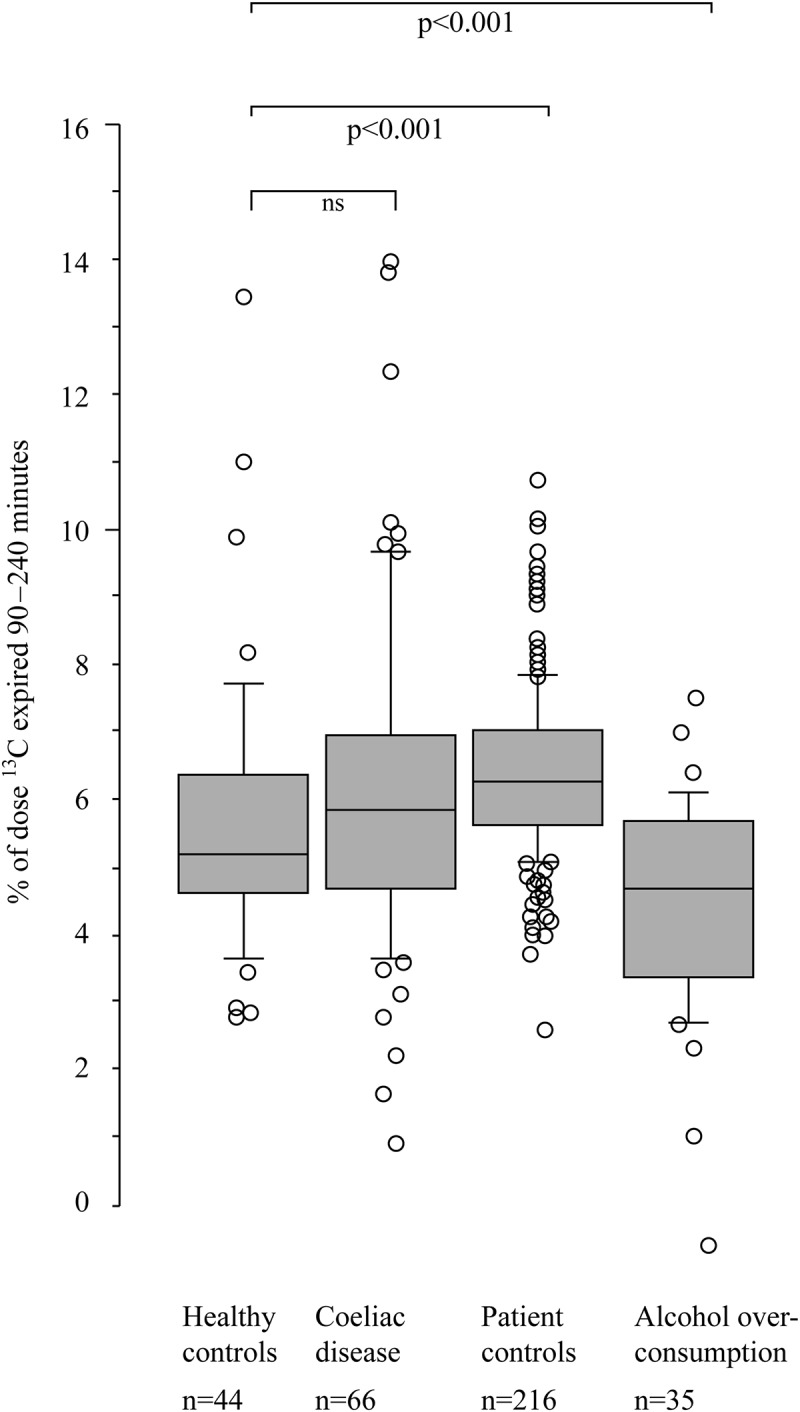
Box plot showing the colonic phase (90–240 min) of ^13^CO_2_ excretion after ingestion of ^13^C-D-xylose in different patient groups. The results are expressed as area under the curve (percentage of dose ^13^C-recovery) for the last 150 min of the test, hence showing total amount of ^13^C-recovery. During this phase, the patients with alcohol overconsumption differ from healthy controls (*p* < 0.001), while there is no significant difference between the other three groups, respectively.

The breath test curves for all four groups are presented in Figure 1. During the small intestinal phase (0–60 min), the alcohol overconsumption group and the coeliac disease group showed a pattern of malabsorption that significantly separated them from all the other groups based on calculations of area under the curve of the first 60 min (*p* < 0.001, Figure 2). No significant difference was found between the alcohol overconsumption group and the coeliac disease group (*p* = 0.22). During the colonic phase (90–240 min), the alcohol overconsumption group differed from the other three groups with regard to total amount of exhaled ^13^CO_2_. The alcohol overconsumption group had a significantly lower ^13^CO_2_ excretion during this phase as compared to the coeliac disease group (*p* < 0.001), the patient control group (*p* < 0.001) and healthy control group (*p* = 0.005), respectively (Figure 3).

## Discussion

In the present study, we explored the effects of chronic alcohol overconsumption by evaluating gastrointestinal function as assessed by D-xylose breath test results. We found that chronic alcohol overconsumption seems to cause malabsorption comparable to patients with untreated coeliac disease, and may alter gut microbial metabolism.

During the small intestinal phase (0–60 min), the breath test results of patients with alcohol overconsumption and coeliac disease were comparable, and these two groups differed from the other groups, indicating malabsorption. Malabsorption is a known problem in patients with coeliac disease,[21] and has previously been shown in patients with alcohol overconsumption.[12,22] Hope et al. [15] have previously reported a possible explanation for this finding, demonstrating a reduced surface of the intestinal epithelium by electron microscopical examination of duodenal biopsies from patients with alcohol overconsumption. Our results thus confirm previous knowledge regarding malabsorption in patients with alcohol overconsumption and coeliac disease.

During the colonic phase (90–240 min), patients with alcohol overconsumption differed from the other three groups, with significantly less ^13^CO_2_ excretion in the period where the unabsorbed part of the test meal is expected to be located within the colon. Several previous studies on the effects of alcohol on the gut microbiota have shown that alcohol abuse affects the composition of the gut microflora. Short- and long-term alcohol abuse seem to create a state of dysbiosis, both in humans and in animal models.[23–25] In general, the balance between the colonic bacteria is affected in a pro-inflammatory manner, with increased amounts of Proteobacteria at the expense of anti-inflammatory bacteria like Bacteroidetes.[23] The effect of alcohol on gut microbial function has also been demonstrated through examinations on short-chain fatty acids (SCFAs), which are end products of colonic fermentation. Patients with a high consumption of alcohol seemingly have less total SCFA levels, but higher levels of acetic acid.[26] An alcohol-induced dysbiosis may affect the body through ‘leakage’ of endotoxins from the bowel to the blood.[23] The main part of the gut microbiota is under normal circumstances situated within the colon.[27] However, a high consumption of alcohol may also affect the small intestine, creating bacterial overgrowth.[28,29] Although several microflora-associated consequences have been demonstrated in relation to alcohol intake, the pathophysiological explanation on how alcohol acts to affect the gut microflora is under debate.[4] Our hypothesis is that dysbiosis may explain the findings of an altered colonic phase in the group of patients with alcohol overconsumption in the present study.

Some limitations to the study design and the breath test itself must be addressed. The retrospective design implies that we made no standardized test-protocol in advance of the inclusion of patients, which also implies that the only test all participants went through was the breath test. Other inquiries were made within a clinical setting, individually for each patient. In order to gather the most possible information on the patients’ diagnoses, we reviewed patient files thoroughly on a regular basis up to the point of analysing the data. Consequently, only 10 patients remained without diagnosis.

One of the advantages of the breath tests, namely their non-invasive character, is simultaneously one of the weaknesses of these tests. The pathways through which the test meal is metabolized, within the liver and the colon, is not completely understood. Moreover, the milieu, in which the test meal is transported and metabolized, is impossible to standardize. Consequently, a variation in bodily functions, like intestinal transit time or hepatic metabolism of the test meal, or variation in the nutritional context in which the D-xylose is metabolized, might theoretically affect the results. In analysing the data, we applied an estimated OCTT of 90 min. Breath tests using liquid meals are more uncertain than solid meal-tests regarding OCTT, with large inter- as well as intra-individual differences.[30] There seems however to be a certain consensus that OCTT for liquid meals, like lactulose, is around 90–100 min.[13,31] When analysing the colonic phase, we therefore chose to apply an OCTT of 90 min, in order to be certain to separate the time the test meal spends in the colon from the small intestinal phase. However, a combination of a prolonged OCTT and malabsorption in patients with alcohol overconsumption may have created similar results as in the present study. If the test meal, due to a prolonged small intestinal phase, spends greater parts of the test time in an area where it is not absorbed, nor digested by bacteria, then both the first and latter part of the curve would lie beneath a normal curve. However, the effect of alcohol on OCTT is not clear, since alcohol overconsumption has been found both to prolong [31,32] and/or shorten the OCTT.[33] Furthermore, the OCTT has to be severely prolonged in order to create results like the present breath test results. A decline in liver function, as seen in decompensated liver cirrhosis (regardless of cause), might affect the D-xylose test results.[34,35] This discovery was one of the corner stones in the development of the D-xylose test as a breath test,[8] as cirrhotic patients differed from patients with adequate liver function. However, the results of patients with compensated liver failure were not affected.[34,36] Consequently, patients with clinical and/or biochemical signs of decompensated liver failure were not included in our study. Malnutrition is a known problem in patients with a prolonged over-consumption of alcohol. Patients with high alcohol consumption tend to base larger parts of the energy intake on alcohol-containing liquids. In addition, regarding solid meals, the replacement of healthy food in favour of unhealthy ones is also a known problem.[37] Whether malnutrition directly affects the intestinal handling of the test meal is unclear. It may however lead to alterations of the gut microbiota.[27] As mentioned above, alcohol overconsumption may increase luminal levels of acetic acid.[26] In an industrial setting, the presence of acetic acid may inhibit metabolism of xylose.[38] The presence of ethanol itself may also impair the metabolism of xylose.[39,40] However, whether these phenomena also occur *in vivo* is unclear.

In conclusion, alcohol overconsumption seems to be associated with small intestinal malabsorption, corresponding with previous findings. Furthermore, a high alcohol intake may cause alterations in the composition and/or functions of the colonic microflora that are evident by the ^13^C-D-xylose breath test results. The relation between alcohol overconsumption and gut microflora alterations should be investigated further.

## References

[CIT0001] Bode C, Bode JC. (1997). Alcohol’s role in gastrointestinal tract disorders. Alcohol Health Res World.

[CIT0002] Gao B, Bataller R (2011). Alcoholic liver disease: pathogenesis and new therapeutic targets. Gastroenterology.

[CIT0003] Lucey MR, Mathurin P, Morgan TR (2009). Alcoholic hepatitis. N Engl J Med.

[CIT0004] Vassallo G, Mirijello A, Ferrulli A (2015). Review article: alcohol and gut microbiota - the possible role of gut microbiota modulation in the treatment of alcoholic liver disease. Aliment Pharmacol Ther.

[CIT0005] Leclercq S, Matamoros S, Cani PD (2014). Intestinal permeability, gut-bacterial dysbiosis, and behavioral markers of alcohol-dependence severity. Proc Natl Acad Sci U S A.

[CIT0006] Midtvedt T, Bjorneklett A, Carlstedt-Duke B, Wostman BS (1985). The influence of antibiotics upon microflora associated characteristics in man and animals. Germfree research; Microflora control and its application to the biochemical sciences. Progress in clinical and biological research.

[CIT0007] King CE, Toskes PP (1986). Comparison of the 1-gram [14C]xylose, 10-gram lactulose-H2, and 80-gram glucose-H2 breath tests in patients with small intestine bacterial overgrowth. Gastroenterology.

[CIT0008] Pitkanen E (1977). The conversion of D-xylose into D-threitol in patients without liver disease and in patients with portal liver cirrhosis. Clin Chim Acta.

[CIT0009] Craig RM, Murphy P, Gibson TP (1983). Kinetic analysis of D-xylose absorption in normal subjects and in patients with chronic renal failure. J Lab Clin Med.

[CIT0010] Craig RM, Ehrenpreis ED (1999). D-xylose testing. J Clin Gastroenterol.

[CIT0011] Tveito K, Brunborg C, Sandvik L (2008). 13C-xylose and 14C-xylose breath tests for the diagnosis of coeliac disease. Scand J Gastroenterol.

[CIT0012] Hope H, Skar V, Sandstad O (2012). Small intestinal malabsorption in chronic alcoholism: a retrospective study of alcoholic patients by the (1, 4)C-D-xylose breath test. Scand J Gastroenterol.

[CIT0013] Simren M, Stotzer PO (2006). Use and abuse of hydrogen breath tests. Gut.

[CIT0014] Tveito K, Brunborg C, Bratlie J (2010). Intestinal malabsorption of D-xylose: comparison of test modalities in patients with celiac disease. Scand J Gastroenterol.

[CIT0015] Hope HB, Tveito K, Aase S (2010). Small intestinal malabsorption in chronic alcoholism determined by 13C-D-xylose breath test and microscopic examination of the duodenal mucosa. Scand J Gastroenterol.

[CIT0016] Hope HB, Medhus AW, Sandstad O (2011). Reduced 13C-D-xylose absorption in alcoholics is more likely caused by alterations in small intestinal mucosa than delayed gastric emptying. Scand J Gastroenterol.

[CIT0017] Schoeller DA, Klein PD, MacLean WC (1981). Fecal 13C analysis for the detection and quantitation of intestinal malabsorption. Limits of detection and application to disorders of intestinal cholylglycine metabolism. J Lab Clin Med.

[CIT0018] Haycock GB, Schwartz GJ, Wisotsky DH (1978). Geometric method for measuring body surface area: a height-weight formula validated in infants, children, and adults. J Pediatr.

[CIT0019] Hirakawa M, Iida M, Kohrogi N (1988). Hydrogen breath test assessment of orocecal transit time: comparison with barium meal study. Am J Gastroenterol.

[CIT0020] Bond JH, Levitt MD, Prentiss R (1975). Investigation of small bowel transit time in man utilizing pulmonary hydrogen (H2) measurements. J Lab Clin Med.

[CIT0021] Rubio-Tapia A, Hill ID, Kelly CP (2013). ACG clinical guidelines: diagnosis and management of celiac disease. Am J Gastroenterol.

[CIT0022] Green PH (1983). Alcohol, nutrition and malabsorption. Clin Gastroenterol.

[CIT0023] Patel S, Behara R, Swanson GR (2015). Alcohol and the Intestine. Biomolecules.

[CIT0024] Mutlu E, Keshavarzian A, Engen P (2009). Intestinal dysbiosis: a possible mechanism of alcohol-induced endotoxemia and alcoholic steatohepatitis in rats. Alcohol Clin Exp Res.

[CIT0025] Mutlu EA, Gillevet PM, Rangwala H (2012). Colonic microbiome is altered in alcoholism. Am J Physiol Gastrointest Liver Physiol.

[CIT0026] Xie G, Zhong W, Zheng X (2013). Chronic ethanol consumption alters mammalian gastrointestinal content metabolites. J Proteome Res.

[CIT0027] Sekirov I, Russell SL, Antunes LC (2010). Gut microbiota in health and disease. Physiol Rev.

[CIT0028] Bode C, Kolepke R, Schafer K (1993). Breath hydrogen excretion in patients with alcoholic liver disease–evidence of small intestinal bacterial overgrowth. Z Gastroenterol.

[CIT0029] Hauge T, Persson J, Danielsson D (1997). Mucosal bacterial growth in the upper gastrointestinal tract in alcoholics (heavy drinkers). Digestion.

[CIT0030] Gasbarrini A, Corazza GR, Gasbarrini G (2009). Methodology and indications of H2-breath testing in gastrointestinal diseases: the Rome Consensus Conference. Aliment Pharmacol Ther.

[CIT0031] Papa A, Tursi A, Cammarota G (1998). Effect of moderate and heavy alcohol consumption on intestinal transit time. Panminerva Med.

[CIT0032] Addolorato G, Montalto M, Capristo E (1997). Influence of alcohol on gastrointestinal motility: lactulose breath hydrogen testing in orocecal transit time in chronic alcoholics, social drinkers and teetotaler subjects. Hepatogastroenterology.

[CIT0033] Keshavarzian A, Iber FL, Dangleis MD (1986). Intestinal-transit and lactose intolerance in chronic alcoholics. Am J Clin Nutr.

[CIT0034] Shamma’A MH, Ghazanfar SA (1960). D-xylose test in enteric fever, cirrhosis, and malabsorptive states. Br Med J.

[CIT0035] Finlay JM, Wightman KJ (1958). The xylose tolerance test as a measure of the intestinal absorption of carbohydrate in sprue. Ann Intern Med.

[CIT0036] Christiansen PA, Kirsner JB, Ablaza J (1959). D-Xylose and its use in the diagnosis of malabsorptive states. Am J Med.

[CIT0037] Breslow RA, Guenther PM, Juan W (2010). Alcoholic beverage consumption, nutrient intakes, and diet quality in the US adult population, 1999-2006. J Am Diet Assoc.

[CIT0038] Sakihama Y, Hasunuma T, Kondo A (2015). Improved ethanol production from xylose in the presence of acetic acid by the overexpression of the HAA1 gene in Saccharomyces cerevisiae. J Biosci Bioeng.

[CIT0039] du Preez JC, Bosch M, Prior BA (1987). Temperature profiles of growth and ethanol tolerance of the xylose-fermenting yeasts Candida shehatae and Pichia stipitis. Appl Microbiol Biotechnol.

[CIT0040] Zhao L, Yu J, Zhang X (2010). The ethanol tolerance of Pachysolen tannophilus in fermentation on xylose. Appl Biochem Biotechnol.

